# Inescapable Fibrosis: The Development of Desquamative Interstitial Pneumonia Post-Lung Transplantation Performed for a Patient with Idiopathic Pulmonary Fibrosis

**DOI:** 10.1155/2023/1737309

**Published:** 2023-04-12

**Authors:** Paul Lilburn, Divya Pillutla, Vanathi Sivasubramaniam, Marshall Plit

**Affiliations:** ^1^University of New South Wales, Kensington, NSW 2052, Australia; ^2^Macquarie University Hospital, 3 Technology Place, North Ryde, NSW 2109, Australia; ^3^Department of Respiratory Medicine, Prince of Wales Hospital, Randwick, NSW 2031, Australia; ^4^St. Vincent's Hospital, 390 Victoria Street, Darlinghurst, NSW 2010, Australia

## Abstract

Interstitial lung disease is characterised by a combination of cellular proliferation, inflammation of the interstitium and fibrosis within the alveolar wall. A 58-year-old man was referred for lung transplantation after developing worsening dyspnoea and progressive hypoxaemic respiratory failure from idiopathic pulmonary fibrosis. Three years later, he developed desquamative interstitial pneumonia in his transplanted lungs, and despite augmentation of immune suppression, he had a progressive decline in his lung function and exercise capacity. Interestingly, in our case, the histopathology obtained post transplant strongly goes against the recurrence of usual interstitial pneumonia/idiopathic pulmonary fibrosis; rather, two separate interstitial disease processes have been identified.

## 1. Introduction

In 1952, three principal groups of infiltrative lung disease emerged, namely, sarcoidosis, environmental lung diseases, and chronic interstitial pneumonias. Carrington et al. in 1978 then described the natural history of two distinct types of interstitial lung disease (ILD)—usual interstitial pneumonia (UIP) and desquamative interstitial pneumonia (DIP) [1]. Initially, it was debated whether these were merely the early and later stages of a single disorder; however, they were later accepted as two distinct pathologies. Further classification has evolved with a broadened understanding of the differing clinical entities categorised as interstitial lung disease (ILD) [2]. The now large family of interstitial lung diseases is characterised by a combination of cellular proliferation, inflammation of the interstitium, and fibrosis within the alveolar wall.

Idiopathic pulmonary fibrosis (IPF) is the most common ILD with unknown clinical origin. The characteristic morphological feature of IPF is a steroid-resistant disease which pertains to a relatively poor prognosis. The prevalence of IPF is reported to range ~30 cases per 100,000 adults. The burden of disease is on the increase with hospitalisations and deaths from IPF rising [3]. This, as well as other factors, has led to a growing number of lung transplantations occurring for idiopathic pulmonary fibrosis. DIP has a more favourable time course than IPF and is comparatively steroid responsive with known environmental triggers that are well described. The most common environmental trigger is tobacco smoking leading to intracytoplasmic coloured granules in alveolar macrophages, considered a characteristic of “smoker's macrophages” in DIP [4].

Other aetiologies for DIP include infectious agents, occupational exposures, and haematological disorders [5].

In this report, we describe a case of a gentleman who was diagnosed with IPF and subsequently underwent bilateral single sequential lung transplantation later to develop DIP, the cause of which remains unclear.

## 2. Case Report

**Figure 1:**
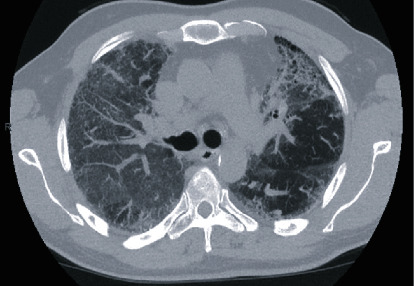
CT chest pretransplant (28/04/2015) demonstrating a UIP pattern of IPF.

A 58-year-old man was referred to see a respiratory physician in a tertiary centre in Sydney, NSW in June 2015 after developing worsening dyspnoea and hypoxaemic respiratory failure. He was an ex smoker with no known previous exposures. His past medical history was significant for concomitant moderate pulmonary hypertension. In June 2015, his baseline FEV1 was 1.34 L (40% predicted), FVC 1.61 L (39% predicted), TLC 2.43 L (37% predicted), and a DLCO of 5 mL/min/mmHg (16% predicted). Investigations included a negative autoimmune screen and computed tomography (CT) imaging which demonstrated interstitial thickening, honeycombing, and traction bronchiectasis (Figure 1) suggestive of a radiological UIP pattern.

**Figure 2:**
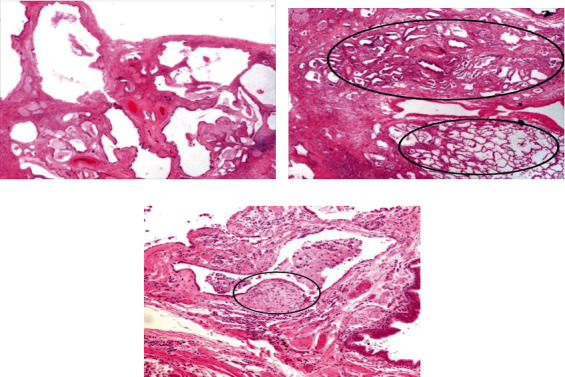
(a) Histopathology specimens demonstrating extensive areas of established fibrosis and extensive remodelling producing honeycombing. (b) Histopathology section demonstrating subpleural and paraseptal predominance of the fibrosis with small areas of relative preservation without fibrosis. (c) Histopathology section demonstrating the presence of fibroblast foci.

A video-assisted-thoracoscopy (VATS) biopsy was performed. Histopathology specimens demonstrated extensive areas of established fibrosis and extensive remodelling producing end-stage lung disease with honeycombing (Figure 2), especially in a subpleural and septal distribution, with small areas of relative preservation without fibrosis (Figure 2(b)) and scattered fibroblastic foci (Figure 2(c)), all consistent with UIP.

The consensus diagnosis at an ILD multidisciplinary team meeting was IPF which was subsequently treated with pirfenidone and the patient referred for lung transplantation.

Our patient underwent a bilateral single sequential lung transplant on the 1^st^ of October 2016. The donor was a 69-year-old female, non smoker. Donor ischemic time right lung was 2 hours 40 minutes and left lung was 3 hours 36 minutes. Twelve hours post transplant, the patient was taken back to the theatre to repair a parenchymal tear in the right lung. Our patient had a primary graft dysfunction grade 3 at 24 hours after transplantation. He required venovenous extracorporeal membrane oxygenation for a duration of 6 days and remained intubated on ventilatory support for a further 3 days.

Post transplant, our patient was given standard immunosuppression as per the St. Vincent's Hospital lung transplant protocol; a tacrolimus infusion at target trough levels of 12-15 mg/ml, intravenous (IV) methylprednisolone, subsequently weaned down to oral twice daily doses of prednisolone 1 mg/kg, IV mycophenolate 1500 mg twice daily, azithromycin for bronchiolitis obliterans syndrome (BOS) prophylaxis, sulfamethoxazole/trimethoprim 800 mg/160 mg once daily for pneumocystis jirovecii pneumonia (PJP) prophylaxis, and amphotericin B nebulised as a preventative strategy against anastomotic fungal infection.

Empirical antimicrobial therapy was IV piperacillin-tazobactam 4.5 g three times daily, de-escalated to cefotaxime 1 g three times daily.

Respiratory viral polymerase chain reaction (PCR) testing on the donor lung swabs detected influenza-A, and so our patient also completed a 5-day course of oseltamivir 75 mg twice daily.

As per the St. Vincent's Hospital post-lung transplant surveillance program, a bronchoscopy and bronchoalveolar lavage were performed at day 1 and day 7 with transbronchial biopsies performed at weeks 3, 6, and 12. No evidence of acute lung allograft rejection was seen (A0B0). The week 12 post-transplant transbronchial biopsy in December 2016 showed changes consistent with organising pneumonia with type 2 pneumocyte hyperplasia and occasional fibroblastic foci. Spirometry at this time was FEV1 2.09 L.

Our patient had chronic rhinovirus positivity on bronchoalveolar lavage PCR testing. Two consecutive spirometry results demonstrated that his mean best FEV1 post transplant was 2.25 L (88.8%) on the 27^th^ of July 2017 (ten months post-transplant).

On the 14^th^ of November 2017, our patient had a drop in lung function to FEV1 2.0 L, which prompted further investigation. A computer tomography pulmonary angiogram (CTPA) displayed subpleural fibrosis, and further investigation with transbronchial biopsy demonstrated evidence of focal organising pneumonia with no acute cellular rejection (A0B0). This was treated with oral prednisone pulse of 1 mg/kg (35 mg) in 2 divided doses weaning by 5 mg every 2 days until a baseline dose of 15 mg twice daily after which spirometry improved to a FEV1 of 2.23 L in December 2017.

**Figure 3:**
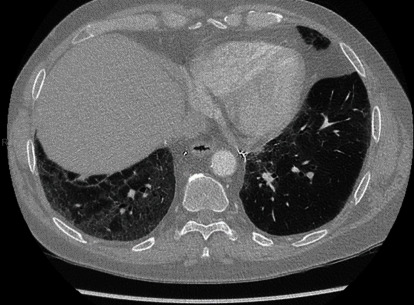
CT chest imaging 2 years (12/09/2018) posttransplant.

Two years post-transplant, in September 2018, follow-up CT chest imaging demonstrated ground glass opacification involving mainly the lower lobes, greater on the right than the left (Figure 3).

**Figure 4:**
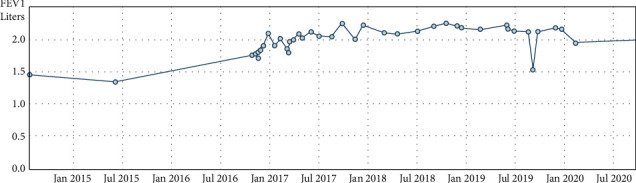
Graph of our patient's FEV1 trend from pretransplant in June 2015 to posttransplant up to July 2020.

At this point, lung function remained stable (Figure 4), FEV1 2.22 L, therefore, no further treatment was initiated.

On the 29^th^ of May 2019, the patient was admitted to the hospital with progressively worsening dyspnoea. A full formal lung function was performed which demonstrated a restrictive ventilatory defect with a severe impairment of the gas transfer factor. Arterial blood gas analysis revealed moderate hypoxaemia with a respiratory alkalosis. Prednisolone dose was increased from regular 7.5 mg daily to 30 mg twice daily during admission.

During May 2019 admission, our patient underwent a transbronchial biopsy of the right middle lobe from which histopathology showed focal organising fibrosis with a prominent accumulation of macrophages in alveolar spaces. Lung microbiology from bronchoalveolar lavage demonstrated the presence of *Aspergillus fumigatus* which was treated with oral itraconazole. No fungal growth had been detected prior to this. Repeated serology had not detected cytomegalovirus (CMV) viraemia at a significant level. Subsequent VATS wedged biopsies from the left upper and lower lobes taken on June 7^th^, 2019, exhibited features of desquamative interstitial pneumonia (DIP) on histology.

**Figure 5:**
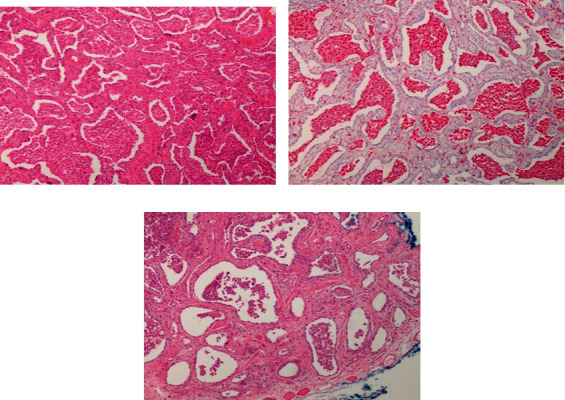
(a) Histopathology showing diffuse, uniform macrophage accumulation within alveolar spaces. (b) Histopathology highlighted on immunochemistry for CD68. (c) Histopathology demonstrating focal interstitial thickening and inflammatory changes.

The sections of the peripheral lung from both lobes showed a diffuse alveolar filling defect with the expansion of the alveolar spaces by numerous alveolar macrophages, a proportion of which contained haemosiderin pigment (Figure 5(a)). These cells were highlighted on an immunoperoxidase stain for CD68 (Figure 5(b)). This was accompanied by focal regions of interstitial fibrosis with the thickening and early distortion of the alveolar septae (Figure 5(c)), with multifocal regions of type 2 pneumocyte hyperplasia and small foci of megakaryocytes, as well as mild interstitial infiltrate within the alveolar septae that included scattered eosinophils but no significant lymphocytic aggregates or prominent germinal centres all consistent with DIP.

Our patient was discharged on the 12^th^ of June 2019 with the following immunosuppression, mycophenolate 1500 mg twice daily, prednisolone 7.5 mg daily, and tacrolimus 1 mg daily. He was subsequently followed up in the lung transplant clinic on the 14^th^ of June after diagnosis of DIP when his immunosuppression was changed to azathioprine 125 mg at night and cyclosporin 100 mg twice daily with prednisolone continued at 7.5 mg daily.

In summary, our patient had IPF in his native lungs in 2015 and underwent a successful bilateral lung transplant in October 2016 with the anticipated improvement in his lung function. Unfortunately, in 2019, he developed DIP in his transplanted lungs with an ongoing gradual decline in his lung function and exercise capacity despite augmentation to his immune suppression.

Follow-up of our patient from electronic medical records (EMR) showed that his pulmonary function tests (PFT) and exercise tolerance continued to deteriorate. Our patient is now oxygen-dependent. The gentleman is currently being considered for workup for a second lung transplant operation.

We believe this to be a rare and unusual process where he acquired a different form of interstitial process from his native lung disease after a lung transplant.

## 3. Discussion

To our knowledge, this is the first reported case of a patient developing DIP post-lung transplant when the initial diagnosis was in fact IPF, UIP pattern.

DIP is a pathology characterised by diffuse filling of alveolar spaces with macrophages followed by interstitial inflammation and fibrosis. There is a 2 : 1 male predominance cited in the literature [6]. Hellemons et al. performed a systematic review of published cases of DIP. Their data set comprised 294 cases of DIP. A mean age of presentation of 40-50 years old (range 16-79 years) was found. The strongest aetiological agent identified was tobacco smoking. However, the correlation was not as strong as seen in other smoking-related ILDs (e.g., Langerhans cell histiocytosis or respiratory bronchiolitis-interstitial lung disease). 70% were active smokers at the time of diagnosis, and 19% of their cohort were recorded as never smokers [7].

A smoking history was not identified in the donor. Medications have been identified as potential triggers of DIP, namely, nitrofurantoin, sulfasalazine, and sirolimus [7]. A case report from 2007 describes a patient with a renal transplant being treated with sirolimus developing DIP [8]. Our patient was not treated with a mammalian target of rapamycin (mTOR) inhibitor nor any other agents reported in the literature. Autoimmune diseases such as systemic sclerosis, rheumatoid arthritis, and systemic lupus erythematosus have also been implicated in the establishment of DIP.

Exposure to copper, beryllium, and nylon filaments has also been described. Infectious agents associated with the disease include *Aspergillus*, Hepatitis C, and CMV [9]. Our patient's occupational history was unremarkable for any significant exposures. We do not have data on environmental exposures of the donor other than tobacco. Bronchoalveolar lavage results were positive for *Aspergillus* in our case which may be of relevance. Treatment with azole therapy does not appear to have altered the trajectory of the disease in our patient.

The principal radiological finding in DIP is diffuse basal predominant ground glass opacification. Other high-resolution computer tomography (HRCT) features reported are irregular reticulation, traction bronchiectasis, and cysts (ranging from minor cysts to honeycombing). Interestingly, in our patient, radiology showed lower lobe predominance. Histopathology often exhibits alveolar infiltration with pigmented (usually light brown) macrophages and characteristics suggestive of a fibrotic nonspecific interstitial pneumonia (NSIP) pattern such as the diffuse widening of alveolar septa. Sporadic eosinophils can also be seen. Toward late-stage disease, fibrosis around the alveolar interstitium can occur. Unlike usual interstitial pneumonia, however, fibroblastic foci are almost never seen. Multinucleated giant cells can also be reported.

Avoidance of the specific exposure is the most important intervention for DIP. Treatment of DIP is predominantly with corticosteroids; 91% of patients received this as their initial therapy in one cohort [7]. Steroid treatment duration was varied from weeks to months. Diken et al. suggest that initial steroid therapy would start from 40-60 mg daily for 6 weeks with subsequent tapering and cessation of treatment over 6-9 months. Other therapies described include macrolides, azathioprine, ribavirin, chloroquine, and cyclophosphamide [5, 7, 10]. No response to treatment is reported to be rare [7]. Our patient was already on long-term corticosteroid therapy, and following the discovery of the disease process, his immunosuppressive regimen was changed.

Recurrence of ILD in lung transplantation has been previously described. A study which looked at 1394 patients at 6 United States transplant centres found that the most common ILD to recur was sarcoidosis, roughly 1% of their cohort [11]. Arboleda et al. describe a case of polymyositis-associated lung disease in a 15-year-old girl who died nine months post transplant. Portmortem examination revealed advanced fibrosis with a UIP pattern which the medical team and authors deemed to be suggestive of disease recurrence [12]. Three cases of DIP recurrence post transplant have been reported. Barberis et al. describe a 49-year-old female treated with a right single lung transplant for DIP. One-month post transplant bronchiolitis obliterans organising pneumonia was seen on transbronchial biopsies performed for a drop in lung function. The patient succumbed to the disease 110 days post transplant despite a pulse of methylprednisolone [13]. A 50-year-old woman treated with a single lung transplant for DIP (secondary to heavy cigarette smoking) developed recurrence after only 1 month post-lung transplantation. She developed worsening infiltrates in the transplanted lung. The patient underwent a thoracoscopic lung biopsy—a light microscopic examination of hematoxylin and eosin-stained sections revealed findings consistent with her underlying DIP. She died of respiratory failure 8 months post transplant. An autopsy concluded that her death was caused by the recurrence of her primary ILD in the transplanted lung [14]. A year later, DIP recurrence post transplant was described in the European Respiratory Journal. A 37-year-old patient diagnosed with DIP in 1978 underwent a single lung transplant in 1991. One year following transplantation, DIP was suspected with ground glass opacities seen basally on the transplanted lung. Transbronchial lung biopsies performed did not detect any evidence of acute or chronic rejection, and bronchoalveolar lavage was negative for infectious aetiologies. The patient recovered with treatment with high-dose corticosteroids.

Notably, however, this patient did not undergo a surgical lung biopsy for confirmation of the diagnosis.

In our patient, there is no clinical or radiological evidence of chronic lung allograft dysfunction (CLAD). Verleden et al. describe CLAD as a relentless progressive clinical course with no improvement, whereas our patient had intermittent episodes of being clinically stable, particularly so in the earlier stages [15]. The radiology and histopathology are also not consistent with CLAD for our patient.

The histopathology obtained post transplant strongly goes against the recurrence of IPF. Two separate interstitial disease processes have been identified in our patient. Despite augmentation of immune suppression, progressive DIP developed in our case.

Lung transplantation is a well-established treatment for carefully selected patients with ILD that has proven survival benefit [16]. Mechanisms which initiate the development of an ILD remain poorly understood. Development of a de novo ILD will greatly decrease the benefit of transplantation. Further research is required to discover the factors which promote the development of an underlying ILD.

## Data Availability

The medical imaging and histopathology images used to support the findings of this case report are available from the corresponding authors upon request.
